# Probiotic Supplementation in Children and Adolescents with ADHD: A Systematic Review and Meta-Analysis of ADHD-Related and Emotional–Behavioral Outcomes

**DOI:** 10.3390/nu18142357

**Published:** 2026-07-17

**Authors:** Yizhen Yan, Haotian Wu, Mengke Han, Li Zhao, Shan-Shan Mao

**Affiliations:** 1 Beijing Key Laboratory of Sports Performance and Skill Assessment, Beijing Sport University, Beijing 100084, China; yanyiz@126.com (Y.Y.);; 2 School of Sports Medicine and Rehabilitation, Beijing Sport University, Beijing 100084, China; 13632475969@163.com (H.W.);; 3 Key Laboratory for Performance Training and Recovery of General Administration of Sport, Beijing Sport University, Beijing 100084, China

**Keywords:** ADHD, probiotics, children, adolescents, gut–brain axis, systematic review, meta-analysis

## Abstract

Background: Dysregulation of the microbiota–gut–brain axis has been implicated in attention-deficit/hyperactivity disorder (ADHD), but randomized controlled trial evidence for probiotic supplementation remains inconsistent. Objective: This systematic review and meta-analysis evaluated the effects of probiotic supplementation on ADHD-related clinical outcomes and emotional–behavioral outcomes in children and adolescents. Methods: PubMed, Embase, Cochrane CENTRAL, Web of Science, PsycINFO, and EBSCO were searched from database inception to October 2025. Randomized controlled trials including participants under 18 years of age with DSM-defined ADHD were eligible. Pooled effects were calculated using standardized mean differences (SMDs) or mean differences (MDs) with 95% confidence intervals (CIs) under random-effects models. Results: Nine RCTs involving 482 participants were included, with intervention durations ranging from 8 to 12 weeks. In the broad exploratory synthesis of ADHD-related clinical outcomes, probiotic supplementation did not show a clear benefit (SMD = −0.25, 95% CI: −0.57 to 0.07; *p* = 0.131). Because this analysis combined conceptually heterogeneous instruments, greater interpretive weight was placed on domain-specific analyses. No clear effects were observed for more specific core symptom-related measures, including SNAP-IV inattention scores and CPT-derived attention-related performance measures. In domain-specific exploratory analyses, CPRS total scores showed a small reduction following probiotic supplementation (SMD = −0.33, 95% CI: −0.65 to −0.01; *p* = 0.041), whereas CBCL total scores did not show a statistically significant effect under the random-effects model (MD = −1.93, 95% CI: −6.30 to 2.44; *p* = 0.386). The SNAP-IV hyperactivity finding was statistically significant but was largely driven by a single study and should be interpreted cautiously. Conclusions: Current 8–12-week RCT evidence does not demonstrate a clear benefit of probiotic supplementation for ADHD core symptom-related measures. However, exploratory findings suggest that probiotic supplementation may have potential for improving broad parent-reported emotional–behavioral outcomes, particularly CPRS-assessed behavioral symptoms. This potential benefit should be interpreted cautiously because it was based on a small number of heterogeneous trials and was not consistently supported by CBCL outcomes. Larger, rigorously designed trials are needed to confirm these broad parent-reported behavioral and emotional findings and clarify their clinical relevance.

## 1. Introduction

Attention-deficit/hyperactivity disorder (ADHD) is a common neurodevelopmental condition in children and adolescents, characterized by developmentally inappropriate inattention, hyperactivity, and impulsivity that may lead to persistent functional impairment across academic, social, and family settings [1]. Worldwide, the estimated prevalence of ADHD among children and adolescents is approximately 5.29% [2,3], and symptoms often persist into adulthood, affecting long-term mental health, educational attainment, interpersonal functioning, and quality of life [4,5,6]. Although stimulants such as methylphenidate and amphetamines remain the mainstay of pharmacological treatment [7], a proportion of patients show incomplete symptom control, experience adverse effects such as appetite suppression, sleep disturbance, irritability, or poor tolerability, or have difficulty maintaining long-term adherence. In addition, pharmacological treatment may have limited effects on co-occurring emotional and behavioral difficulties, which are common in children with ADHD. These limitations have encouraged interest in complementary and non-pharmacological strategies that may be used as adjunctive approaches to support broader neurobehavioral regulation and improve treatment acceptability [8].

A growing body of evidence links ADHD to dysregulation of the microbiota–gut–brain axis, a bidirectional communication network connecting the gastrointestinal tract, gut microbiota, immune system, endocrine signaling, and central nervous system [9,10,11,12,13]. The gut microbiota may influence ADHD-related neurobehavioral processes through several interconnected pathways. First, microbial metabolites can affect the availability of neurotransmitter precursors, including tryptophan, and may thereby influence serotonergic, dopaminergic, and γ-aminobutyric acid signaling systems involved in attention, reward processing, impulsivity, and emotional regulation [10,11,14,15]. Second, gut microbial dysbiosis may contribute to low-grade systemic inflammation and altered immune signaling, which have been implicated in neurodevelopmental and behavioral abnormalities [10,11,14]. Third, microbial metabolites such as short-chain fatty acids may influence intestinal barrier integrity, blood–brain barrier function, energy metabolism, microglial activation, and neuroimmune communication [13,16]. Children with ADHD have been reported to show differences in gut microbial composition compared with typically developing peers, including lower microbial diversity and altered abundance of genera involved in dopamine and serotonin metabolism [17,18,19]. These findings provide a biological rationale for examining the gut microbiota as a potential modifiable target in ADHD, although causal relationships remain incompletely established.

Probiotic supplementation has therefore attracted increasing attention as a potential strategy for modulating the microbiota–gut–brain axis in ADHD [20,21,22]. Probiotics are defined as live microorganisms that, when administered in adequate amounts, confer a health benefit on the host [23,24]. Several mechanisms have been proposed through which probiotic supplementation may influence ADHD-related symptoms. Certain *Lactobacillus* and *Bifidobacterium* strains may help restore microbial balance, strengthen intestinal barrier function, and reduce lipopolysaccharide translocation, thereby attenuating systemic inflammation and neuroimmune activation [13,25,26]. Probiotics may also affect short-chain fatty acid production, which is relevant to immune regulation, blood–brain barrier integrity, and microglial function [16]. In addition, probiotic supplementation may influence neurotransmitter-related pathways by modulating tryptophan metabolism and the availability of serotonin, dopamine, and γ-aminobutyric acid [15]. Regulation of hypothalamic–pituitary–adrenal axis activity may further contribute to stress responsivity and broader behavioral and emotional processes [25,26]. These mechanisms are biologically plausible, but their clinical relevance in ADHD remains uncertain.

However, the current clinical evidence remains inconclusive. Some randomized controlled trials (RCTs) have reported potential improvements in ADHD symptom ratings or broad behavioral outcomes after probiotic supplementation [27,28], whereas others have found no significant effects [29,30]. These discrepancies may be explained by differences in participant characteristics, diagnostic criteria, probiotic strains, daily doses, intervention durations, comparator conditions, concomitant medication use, and outcome instruments. In addition, outcome measures vary substantially across trials. Some studies focus on core ADHD symptoms, such as inattention and hyperactivity/impulsivity, whereas others assess broader parent-reported behavioral or emotional–behavioral outcomes. Evidence from other psychiatric populations also suggests that probiotic supplementation may produce heterogeneous and often modest clinical effects, with no consistent or sustained changes in gut microbiota diversity or composition after short-term intervention [31]. Therefore, it remains unclear whether probiotic supplementation affects core ADHD symptoms, broader emotional–behavioral outcomes, or neither.

Despite growing interest in probiotic supplementation for ADHD, several important gaps remain. First, previous evidence has not clearly distinguished between overall ADHD-related clinical outcomes, more specific core symptom-related measures, and broader emotional–behavioral outcomes. Second, the available trials differ substantially in probiotic formulation, dose, treatment duration, comparator condition, concomitant medication use, and outcome measurement, making the overall clinical interpretation challenging. Third, the durability and clinical relevance of any observed behavioral changes remain uncertain, particularly because most trials have short intervention periods and limited follow-up. A systematic synthesis that distinguishes overall ADHD-related outcomes and core symptom-related measures from broader emotional–behavioral outcomes is therefore needed to clarify the current evidence and identify priorities for future research.

Therefore, the present systematic review and meta-analysis aimed to evaluate the effects of probiotic supplementation on ADHD-related outcomes in children and adolescents. Specifically, we assessed its effects on overall ADHD-related clinical outcomes, more specific core symptom-related measures, and broader emotional–behavioral outcomes to clarify whether current randomized controlled trial evidence supports a clinically meaningful role for probiotic supplementation in ADHD.

## 2. Materials and Methods

### 2.1. Study Design and Reporting Standards

**Figure 1:**
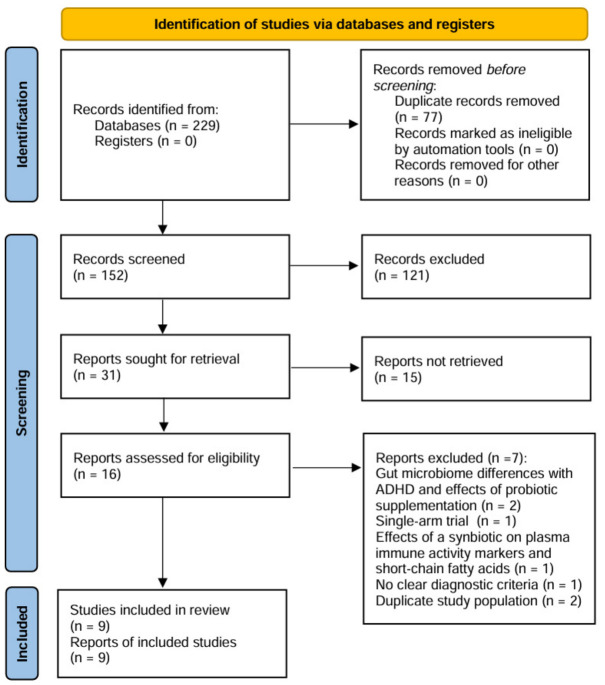
Study selection process following the PRISMA flowchart.

This systematic review and meta-analysis was conducted in accordance with the Preferred Reporting Items for Systematic Reviews and Meta-Analyses (PRISMA) 2020 guidelines [32]. All procedures, including literature search, study selection, data extraction, risk-of-bias assessment, and statistical synthesis, were performed following a predefined methodological framework (Figure 1). The study protocol was prospectively registered in the International Prospective Register of Systematic Reviews (PROSPERO) (Registration No. CRD420251177643) [33].

### 2.2. Literature Search Strategy

A comprehensive literature search was performed in PubMed, Embase, the Cochrane Central Register of Controlled Trials (CENTRAL), Web of Science, PsycINFO, and EBSCO from database inception to October 2025. The search strategy was designed around two core domains: population and intervention. No predefined retrieval restrictions were applied for comparator, outcome, or study design at the search stage.

The rationale for this broad search strategy was that the available literature on probiotic supplementation for ADHD remains limited. In addition, early publications and some clinical studies may not be consistently indexed with relevant subject headings or document type descriptors, such as “clinical trial” or “randomized controlled trial”. Therefore, applying study design filters during the initial database search could have increased the risk of missing potentially eligible studies. To maximize search sensitivity, eligible randomized controlled trials were subsequently identified through staged manual screening according to the prespecified inclusion and exclusion criteria.

The search strategy combined controlled vocabulary terms, where applicable, and free-text terms related to ADHD, children and adolescents, and probiotic-related interventions. No restrictions were applied regarding language, publication date, or publication status. In addition, the reference lists of relevant reviews and included studies were manually screened to identify additional eligible studies not captured by the electronic search. The complete search strategies for all databases are provided in Supplementary File S1.

An example of the complete Web of Science search strategy is provided below: TS = (Attention Deficit Disorder with Hyperactivity OR ADHD OR ADDH OR Attention Deficit Disorders with Hyperactivity OR Attention Deficit Hyperactivity Disorders OR Attention Deficit Hyperactivity Disorder OR Attention Deficit-Hyperactivity Disorder OR Attention Deficit-Hyperactivity Disorders OR Deficit-Hyperactivity Disorder, Attention OR Deficit-Hyperactivity Disorders, Attention OR Disorder, Attention Deficit-Hyperactivity OR Disorders, Attention Deficit-Hyperactivity OR Hyperkinetic Syndrome OR Syndromes, Hyperkinetic OR Attention Deficit Disorder OR Attention Deficit Disorders OR Deficit Disorder, Attention OR Deficit Disorders, Attention OR Disorder, Attention Deficit OR Disorders, Attention Deficit OR Brain Dysfunction, Minimal OR Dysfunction, Minimal Brain OR Minimal Brain Dysfunction) AND TS = (Adolescent OR Adolescents OR Adolescence OR Adolescents, Female OR Adolescent, Female OR Female Adolescent OR Female Adolescents OR Adolescents, Male OR Adolescent, Male OR Male Adolescent OR Male Adolescents OR Youth OR Youths OR Teens OR Teen OR Teenagers OR Teenager OR Children OR Child) AND TS = (Probiotics OR Probiotic OR Prebiotics OR Prebiotic OR Synbiotics OR Synbiotic).

### 2.3. Eligibility Criteria

#### 2.3.1. Inclusion Criteria

(a) Study design: RCTs.
(b) Population: Children or adolescents (<18 years of age) diagnosed with ADHD according to the Diagnostic and Statistical Manual of Mental Disorders (DSM) criteria (DSM-IV-TR or DSM-5) [34].
(c) Intervention: Supplementation with probiotics or synbiotics, administered alone or as an adjunct to standard treatment.
(d) Comparator: Placebo, no intervention, or standard treatment.
(e) Outcomes: Studies reporting quantitative data on eligible ADHD-related clinical outcomes, core symptom-related measures, or broader emotional–behavioral outcomes assessed using validated rating scales, standardized parent-reported behavioral scales, or validated cognitive performance tests.

#### 2.3.2. Exclusion Criteria (Detailed Reasons Are Provided in Supplementary File S2)

(a) Non-randomized, uncontrolled, or single-arm designs.
(b) ADHD diagnosis not based on DSM-IV-TR or DSM-5 criteria, or diagnostic criteria not clearly reported.
(c) Interventions not involving probiotic or synbiotic supplementation, or studies assessing only gut microbiome differences, immune activity markers, or short-chain fatty acids without eligible ADHD-related clinical outcomes.
(d) Duplicate study populations; when multiple reports used overlapping populations, the report with the most complete and eligible outcome data was retained.
(e) Full-text unavailable or insufficient data for quantitative synthesis, including studies without eligible post-intervention ADHD-related clinical outcome data.

### 2.4. Outcome Measures

Outcomes were defined a priori and analyzed as a broad exploratory synthesis of ADHD-related clinical outcomes and as domain-specific exploratory outcomes. To improve interpretability, the outcome categories were defined according to the construct assessed by each instrument.

“Overall ADHD-related clinical outcomes” referred to a broad exploratory synthesis of eligible clinical or performance-based outcomes reported in the included ADHD trials. This category included ADHD-specific symptom scales, such as the ADHD Rating Scale (ADHD-RS) and the Swanson, Nolan and Pelham–IV Rating Scale (SNAP-IV), attention-related performance measures derived from the Continuous Performance Test (CPT), and broader parent-reported behavioral scales, including the Conners’ Parent Rating Scale (CPRS) and the Child Behavior Checklist (CBCL) [35,36,37,38]. Because these instruments assess related but not identical constructs, this overall pooled estimate was interpreted only as a broad exploratory summary and not as evidence for a single unified clinical construct.

“Core symptom-related measures” referred to outcomes more directly aligned with ADHD symptom domains, particularly inattention and hyperactivity/impulsivity. These included SNAP-IV inattention and hyperactivity subscales. CPT-derived indices were considered standardized attention-related performance measures and were analyzed separately because laboratory-based CPT performance does not necessarily reflect real-world ADHD-related impairment.

“Emotional–behavioral outcomes” referred to broad parent-reported behavioral and emotional outcomes assessed using CPRS and CBCL total scores. These scales include domains such as behavioral problems, emotional symptoms, social difficulties, internalizing problems, and externalizing problems, and therefore should not be interpreted as specific measures of emotional regulation or ADHD core symptoms [36,39].

Therefore, domain-specific analyses were used to provide the more clinically interpretable findings.

To reduce selective outcome inclusion and minimize bias related to multiple comparisons, predefined decision rules were applied when an original study reported multiple eligible scales, subscales, or time points within the same outcome domain. First, global composite scores or full-scale total scores were prioritized when available, because they provide a more comprehensive assessment of overall symptom burden and functional impairment. Second, standardized scales that were most frequently reported across the included studies were prioritized to improve comparability across trials and reduce clinical heterogeneity arising from differences in measurement properties. Third, when an original study reported only individual subscales, the subscale most relevant to the corresponding clinical domain was extracted. When multiple post-baseline time points were reported, the immediate post-intervention endpoint was extracted preferentially. The detailed outcome selection and data extraction decisions are provided in Supplementary File S3.

Domain-specific exploratory analyses were conducted according to the outcomes available across the included studies and were based on the same or highly comparable scales whenever possible. These analyses included CPRS total scores, CBCL total scores, SNAP-IV inattention and hyperactivity subscales, and CPT-derived indices, including omission errors, commission errors, and detectability. ADHD-RS was included in the overall ADHD-related clinical outcome analysis but was not analyzed as a separate subgroup because only one included study reported this outcome.

The CPRS family included both the revised long version (CPRS-R:L) and revised short version (CPRS-R:S), as reported in the included trials. Given their comparable constructs but differing scale structures and scoring ranges, CPRS total scores were pooled using standardized mean differences (SMDs). For the CBCL, all included studies reported total scores using the same scale format, allowing the use of mean differences (MDs).

### 2.5. Study Selection, Data Extraction, Risk-of-Bias Assessment, and Certainty of Evidence

#### 2.5.1. Study Selection

All retrieved records were imported into EndNote 20 for duplicate removal, followed by manual verification. Study selection was then conducted independently by two reviewers using a two-stage screening process.

In the first stage, titles and abstracts of all deduplicated records were screened against the prespecified inclusion and exclusion criteria. Each record was classified as “include”, “exclude”, or “uncertain”. Records marked as “include” or “uncertain” by either reviewer were retained for full-text assessment.

In the second stage, the same two reviewers independently assessed the full texts of all potentially eligible studies to determine final inclusion. Reasons for exclusion at the full-text stage were recorded and categorized according to PRISMA 2020 recommendations [32]. Disagreements at both screening stages were first resolved through discussion between the two reviewers. If consensus could not be reached, a third senior reviewer was consulted for final adjudication.

#### 2.5.2. Data Extraction

A standardized data extraction form was developed in advance based on the Cochrane Handbook for Systematic Reviews of Interventions and tailored to the objectives of this review [40]. Extracted information included basic study characteristics, participant characteristics, intervention details, comparator characteristics, and outcome data.

Before formal data extraction, the standardized extraction form was pilot-tested on two included studies to ensure consistency, completeness, and feasibility. The form was refined after pilot testing to clarify ambiguous fields, particularly those related to intervention components, outcome domains, assessment time points, and concomitant medication use. The finalized extraction form was then used for data extraction from all included studies.

Two reviewers independently extracted the following data: author(s), diagnostic criteria, study design, intervention group, control group, sample size (*N*), intervention duration, dose, outcomes, medication use, participant age, and country. These characteristics are summarized in Table 1. Additional information on per-arm sample sizes, sex distribution, baseline severity, ADHD presentation, comorbidities, medication status and dose, adverse events, attrition, adherence, and analysis population is provided in Supplementary File S4. After completion, all extracted data were cross-checked. Discrepancies in numerical or categorical information were resolved by rechecking the original articles. If disagreements remained unresolved, a third senior reviewer made the final decision.

When outcome data were incompletely reported in the article text or tables, attempts were made to contact the corresponding authors by email to obtain original numerical data. If no response was received within two weeks, data were extracted from published figures using WebPlotDigitizer 4.2. Graphically extracted data were independently obtained by two reviewers. The extracted values were accepted only when the inter-rater reliability reached an intraclass correlation coefficient (ICC) of at least 0.95. Inconsistent data points were re-extracted and discussed until consensus was reached. When multiple eligible outcomes or time points were reported within the same study, the predefined decision rules described in Section 2.4 were applied.

#### 2.5.3. Risk-of-Bias Assessment

The methodological quality of included studies was assessed using the Cochrane Risk of Bias 2.0 tool (RoB 2.0) [47]. Risk of bias was evaluated across five domains: bias arising from the randomization process, bias due to deviations from intended interventions, bias due to missing outcome data, bias in measurement of the outcome, and bias in selection of the reported result.

The assessment was conducted at the outcome level rather than only at the study level, because different outcomes within the same study may have different levels of bias risk. Two reviewers independently assessed the risk of bias for each relevant outcome in all included studies. Each domain and the overall risk of bias were judged as “low risk”, “some concerns”, or “high risk” according to RoB 2.0 guidance. Disagreements were resolved through discussion; unresolved disagreements were adjudicated by a senior reviewer. Inter-reviewer agreement for the overall risk-of-bias judgment was calculated using Cohen’s kappa coefficient.

**Figure 2:**
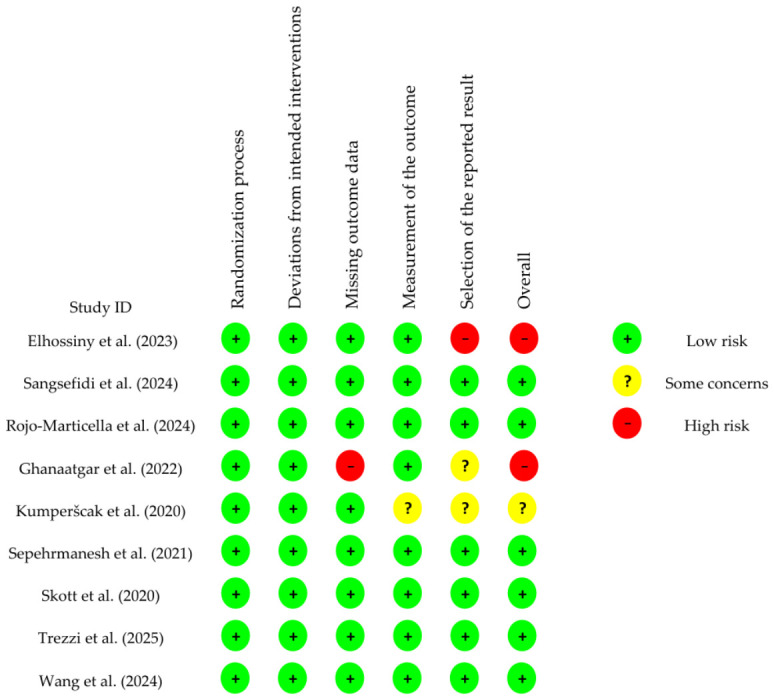
Risk-of-bias summary: authors’ judgments about each risk-of-bias item for each included study [27,28,29,41,42,43,44,45,46].

The risk-of-bias results were visualized using a risk-of-bias summary figure (Figure 2).

#### 2.5.4. Certainty of Evidence Assessment

The certainty of evidence was assessed for the primary pooled outcome, namely overall ADHD-related clinical outcomes, using the Grading of Recommendations Assessment, Development and Evaluation (GRADE) approach in accordance with the Cochrane Handbook [40].

Because all included studies were randomized controlled trials, the certainty of evidence initially started as high. Evidence certainty was then downgraded, where appropriate, based on risk of bias, inconsistency, indirectness, imprecision, and publication bias. The GRADE evidence profile is provided in Supplementary File S5.

### 2.6. Statistical Analysis

All statistical analyses were conducted using STATA version 18.0 (StataCorp, College Station, TX, USA). For continuous outcomes, SMDs or MDs with 95% confidence intervals (CIs) were calculated, as appropriate. SMDs were used when studies assessed comparable constructs using different versions or scoring systems of the similar scale, whereas MDs were used when outcomes were measured using the same scale and scoring metric. For the broad exploratory synthesis of ADHD-related clinical outcomes, SMDs were used to summarize effects across different scales. However, because the included instruments assessed related but not identical constructs, this analysis was not treated as a construct-specific estimate of core ADHD symptom improvement.

A random-effects model was used for all meta-analyses. This decision was made a priori because the included studies showed inherent clinical and methodological heterogeneity in participant characteristics, probiotic strains and formulations, daily doses, intervention durations, concomitant medication use, comparator conditions, and outcome assessment tools. Compared with a fixed-effect model, the random-effects model accounts for both within-study sampling error and between-study variability, and therefore provides a more conservative and clinically appropriate estimate when there is a presence of expected heterogeneity [40,48]. In addition, because several subgroup analyses included only a small number of studies, statistical tests for heterogeneity, including Cochran’s Q test and the *I*^2^ statistic, may have limited power. Therefore, the choice of statistical model was not based solely on the magnitude or statistical significance of heterogeneity tests.

Statistical heterogeneity was quantified using Cochran’s Q test and the *I*^2^ statistic [49]. *I*^2^ values were interpreted as indicating low, moderate, or substantial heterogeneity when appropriate, but these values were interpreted cautiously when the number of included studies was small. Statistical significance was defined as a two-sided *p* < 0.05. For interpretation of standardized effect sizes, SMDs were interpreted using conventional thresholds: negligible (<0.20), small (0.20 to <0.50), moderate (0.50 to <0.80), and large (≥0.80). This categorization was not applied to MD outcomes because MDs are scale-dependent.

For Skott et al. (2020) [44], SNAP-18 inattention and hyperactivity/impulsivity subscale data were reported as item-level mean scores with interquartile ranges (IQRs), without standard deviations (SDs). Each subscale contained nine items scored from 0 to 3. To harmonize these data with the other included studies, item-level IQRs were first calculated as Q3 − Q1, and item-level SDs were then estimated using the approximation SD ≈ IQR/1.35 [40]. Item-level means and estimated item-level SDs were subsequently multiplied by 9 to convert them into subscale total-score means and SDs. This conversion assumes approximate symmetry of the underlying distribution and was therefore considered an estimated value when interpreting the SNAP-IV subgroup results. All converted values were retained to three decimal places without manual rounding during the calculation process.

For Wang et al. (2024) [46], quantitative outcome data were not fully reported in the article text or tables and were available only in published figures. After attempts to contact the corresponding author for original numerical data were unsuccessful, outcome data were extracted from the published figures using WebPlotDigitizer. Figure-based data extraction was independently performed by two reviewers and cross-checked for accuracy. The influence of this study on the pooled estimates was further evaluated using leave-one-out sensitivity analysis.

Sensitivity analyses were performed for the primary outcome using a leave-one-out approach to assess the influence of individual studies on the pooled estimate. Publication bias was assessed for the primary outcome using funnel plot inspection, Begg’s test, and Egger’s test. Publication bias was not formally assessed for subgroup analyses because the number of included studies in each subgroup was small, making such assessments unreliable.

Forest plots were generated in STATA using the metan command to display individual study effect sizes, 95% CIs, study weights, pooled estimates, and heterogeneity statistics. Leave-one-out sensitivity analyses were performed using the metaninf command. Funnel plots were generated using the metafunnel command and were visually inspected for asymmetry. The PRISMA flow diagram was prepared using the PRISMA 2020 flow diagram template [32]. Risk-of-bias results were visualized using a risk-of-bias summary figure based on the RoB 2.0 assessments [47].

## 3. Results

### 3.1. Broad Exploratory Synthesis of ADHD-Related Clinical Outcomes

A total of nine randomized controlled trials (*n* = 482) were included in the analysis of overall ADHD-related clinical outcomes. Across the included studies, the duration of probiotic supplementation ranged from 8 to 12 weeks, and different outcome measures were used to assess ADHD-related symptoms or outcomes. Consistent with the prespecified statistical approach, a random-effects model was used for the pooled analysis.

**Figure 3:**
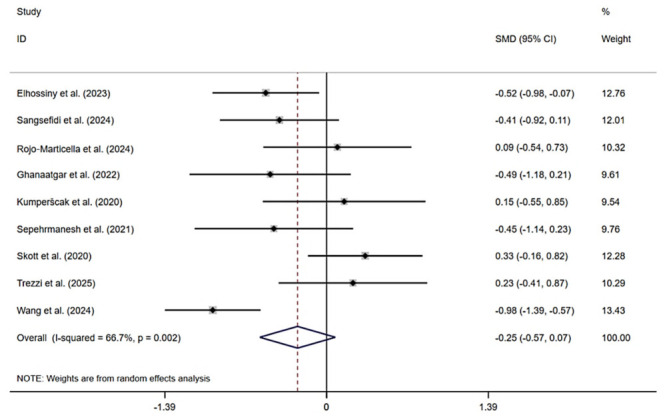
Forest plot of the broad exploratory synthesis of ADHD-related clinical outcomes [27,28,29,41,42,43,44,45,46]. The red dashed vertical line is positioned at the pooled effect estimate (SMD =−0.25).

The pooled analysis showed that probiotic supplementation did not demonstrate a clear benefit for overall ADHD-related clinical outcomes (SMD = −0.25, 95% CI: −0.57 to 0.07; Z = 1.51, *p* = 0.131; Figure 3). Given the conceptual heterogeneity among the included outcome measures, this pooled estimate should be interpreted as a broad exploratory summary of ADHD-related clinical outcomes rather than as a construct-specific estimate of core ADHD symptom improvement. Moderate heterogeneity was observed across studies (*I*^2^ = 66.7%, *p* = 0.002), suggesting variability among the included trials, possibly related to differences in assessment tools, intervention characteristics, and study populations.

The leave-one-out sensitivity analysis, which sequentially omitted each individual study, showed that the 95% confidence intervals of the recalculated pooled effect estimates consistently crossed the null value. This indicates that the conclusion of no statistically significant difference between the intervention and control groups remained stable at the statistical inference level. Although exclusion of individual studies affected the direction or magnitude of the pooled estimate, the overall result remained non-significant (Supplementary File S6, Figure S1).

Publication bias was assessed using funnel plot symmetry as well as Begg’s and Egger’s tests. The funnel plot appeared visually symmetrical, and neither Begg’s test (*p* = 0.4655) nor Egger’s test (*p* = 0.1760) indicated significant publication bias, although the small number of included studies limits the reliability of these assessments (Supplementary File S6, Figure S2).

### 3.2. Domain-Specific Subgroup Analyses

Domain-specific subgroup analyses were conducted according to the outcomes available across the included studies. These analyses included CPRS total scores, CBCL total scores, SNAP-IV inattention and hyperactivity subscales, and CPT-derived indices. Because most subgroup analyses included only two to four studies, these findings were considered exploratory. Formal publication bias assessment and subgroup-level sensitivity analyses were not performed because of the limited number of studies.

#### 3.2.1. CPRS Total Score

Four studies (*n* = 208) were included in the analysis of CPRS total scores, incorporating different versions of the scale (CPRS, CPRS-R:L, and CPRS-R:S). These included Elhossiny et al. (2023) [27], Trezzi et al. (2025) [45], Ghanaatgar et al. (2022) [41], and Sangsefidi et al. (2024) [28]. Because different versions of the CPRS were used across studies, standardized mean differences were used for effect size pooling.

**Figure 4:**
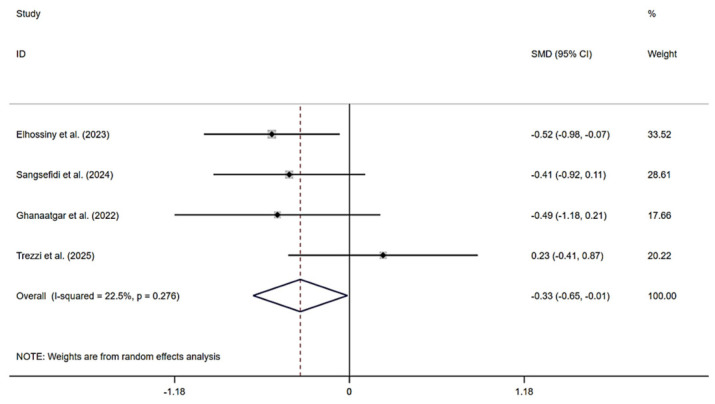
Forest plot of subgroup analysis for total scores on the CPRS series of scales [27,28,41,45]. The red dashed vertical line is positioned at the pooled effect estimate (SMD = −0.33).

Under the random-effects model, probiotic supplementation was associated with a small reduction in CPRS total scores (SMD = −0.33, 95% CI: −0.65 to −0.01; Z = 2.05, *p* = 0.041; Figure 4). This finding suggests a possible improvement in broad parent-reported behavioral symptoms. However, because this subgroup included only four studies and used different versions of the CPRS, the result should be interpreted as exploratory rather than confirmatory.

#### 3.2.2. CBCL Total Score

Three studies (*n* = 146) were included in the analysis of CBCL total scores. Under the random-effects model, probiotic supplementation did not show a statistically significant effect on CBCL total scores (MD = −1.93, 95% CI: −6.30 to 2.44; Z = 0.87, *p* = 0.386; *I*^2^ = 43.7%; Supplementary File S6, Figure S3). Although the pooled estimate favored probiotic supplementation, the confidence interval crossed the null value, indicating uncertainty in the effect estimate.

#### 3.2.3. SNAP-IV Outcomes

Two studies (*n* = 170) were included in the analyses of SNAP-IV inattention and hyperactivity scores. Probiotic supplementation did not show a statistically significant effect on SNAP-IV inattention scores under the random-effects model (MD = 0.29, 95% CI: −2.32 to 2.91; Z = 0.22, *p* = 0.826; *I*^2^ = 76%; Supplementary File S6, Figure S4). The direction of effect differed between Skott et al. (2020) [44] and Wang et al. (2024) [46], which may partly explain the substantial heterogeneity. In addition, the SNAP-18 data from Skott et al. were converted from item-level mean scores with IQRs to estimated total-score means and SDs, which may have introduced additional uncertainty because the conversion assumes approximate distributional symmetry in bounded ordinal symptom scores.

For SNAP-IV hyperactivity scores, the pooled estimate showed a statistically significant difference favoring the control group under the random-effects model (MD = 0.69, 95% CI: 0.39 to 1.00; Z = 4.41, *p* < 0.001; *I*^2^ = 0%; Supplementary File S6, Figure S5). However, this finding was almost entirely driven by Wang et al. (2024) [46], which contributed 98.98% of the weight, whereas Skott et al. (2020) [44] had a very imprecise estimate, with the point estimate in the opposite direction. Therefore, this result should be interpreted cautiously and should not be considered definitive evidence that probiotic supplementation worsens hyperactivity symptoms.

#### 3.2.4. CPT Outcomes

CPT-derived outcomes were reported by two to three studies. No statistically significant effects were observed for commission errors (MD = −0.14, 95% CI: −2.53 to 2.26; Z = 0.11, *p* = 0.91; *I*^2^ = 87.4%; Supplementary File S6, Figure S6), omission errors (MD = −2.18, 95% CI: −5.58 to 1.22; Z = 1.26, *p* = 0.21; *I*^2^ = 78%; Supplementary File S6, Figure S7), or detectability (MD = −0.52, 95% CI: −2.22 to 1.18; Z = 0.60, *p* = 0.55; *I*^2^ = 78.9%; Supplementary File S6, Figure S8).

These findings do not support a clear effect of probiotic supplementation on CPT-derived attention-related performance measures. However, CPT indices should not be interpreted as direct evidence of real-world clinical improvement because laboratory-based attention tasks do not necessarily map directly onto day-to-day ADHD-related impairment.

## 4. Discussion

### 4.1. Principal Findings

This systematic review and meta-analysis included nine randomized controlled trials involving 482 children and adolescents with ADHD. The broad exploratory synthesis of ADHD-related clinical outcomes did not demonstrate a clear benefit of probiotic supplementation. Because this analysis combined instruments assessing related but not identical constructs, including ADHD-specific symptom scales, broad parent-reported behavioral scales, and CPT-derived performance measures, it should not be interpreted as evidence for a single unified clinical construct.

Therefore, greater interpretive weight should be placed on the domain-specific analyses. No clear effects were observed for more specific core symptom-related measures, including SNAP-IV inattention scores and CPT-derived attention-related performance measures. In domain-specific subgroup analyses, CPRS total scores showed a small exploratory reduction following probiotic supplementation, but this signal was not consistently supported by CBCL total scores.

In domain-specific subgroup analyses, CPRS total scores showed a small reduction following probiotic supplementation. However, this exploratory signal was not consistently supported by CBCL total scores, which did not show a statistically significant effect under the random-effects model. Therefore, the current evidence does not support a definitive benefit of probiotic supplementation for broad emotional–behavioral outcomes. The CPRS finding should be interpreted as a preliminary signal in broad parent-reported behavioral symptoms rather than as confirmatory evidence of improvement in specific emotional regulation, behavioral adaptability, or other clearly defined clinical domains.

### 4.2. Interpretation of Differential Effects Across Outcome Domains

#### 4.2.1. Limited Effects on the Broad Exploratory Synthesis and Core Symptom-Related Measures

The broad exploratory synthesis showed no significant improvement in ADHD-related clinical outcomes following probiotic supplementation (SMD = −0.25, *p* = 0.131). Consistently, more specific core symptom-related measures, including the SNAP-IV inattention subscale and CPT-derived attention-related performance measures, also showed no clear improvement. SNAP-IV inattention scores are more closely aligned with core ADHD symptom ratings, whereas CPT-derived indices provide standardized laboratory-based measures of attention-related performance. However, CPT performance should not be equated with real-world ADHD-related impairment or objective clinical improvement.

Several neurobiological and timing-related factors may explain the null findings for core symptom-related outcomes. Core ADHD symptoms are primarily rooted in dysfunctions of fronto–striatal–parietal circuits, particularly involving the prefrontal cortex, striatum, and cerebellum [50,51]. These circuits govern executive functions such as inhibitory control and attentional shifting and rely on fast, precise dopaminergic and noradrenergic signaling [52]. Medications that are effective for core symptoms, such as methylphenidate, act by increasing synaptic catecholamine levels in these circuits [8]. In contrast, probiotics are thought to modulate systemic inflammation, serotonin precursor availability, and HPA axis reactivity [15,53,54,55,56,57,58], pathways that are more closely linked to emotional regulation and stress responses than to the rapid modulation of prefrontal-striatal dopamine transmission. Thus, from a mechanistic perspective, microbiota-targeted interventions may be less likely to directly improve core neurocognitive symptoms [50].

Another important consideration is intervention timing. All included trials administered supplementation during childhood or adolescence (ages 4–18 years) for 8–12 weeks. By this age, the aberrant neurodevelopmental trajectories of ADHD, including delayed cortical maturation and executive function deficits, are already established [59,60]. It remains possible that microbial interventions could have a greater impact if given during early critical windows, before symptoms fully manifest. For example, Pärtty et al. found that giving *Lactobacillus rhamnosus GG* during infancy was associated with a lower risk of ADHD diagnosis at school age [61]. Therefore, the lack of improvement observed in the present analysis does not rule out potential benefits of earlier or longer supplementation. However, within the scope of the available evidence—short-term supplementation in already diagnosed children and adolescents—probiotic supplementation has not demonstrated a clear effect on specific core symptom-related measures.

In summary, the combination of mismatched mechanistic targets and a potentially missed developmental window may partly explain the observed null findings for specific core symptom-related measures [50].

#### 4.2.2. Effects on Broad Parent-Reported Behavioral and Emotional Outcomes

In contrast to the null findings for core ADHD symptom measures, CPRS total scores showed a small reduction following probiotic supplementation (SMD = −0.33, *p* = 0.041). However, CBCL total scores did not show a statistically significant effect under the random-effects model (MD = −1.93, *p* = 0.386). Therefore, the evidence for broad parent-reported emotional–behavioral outcomes should be interpreted as limited and inconsistent rather than as evidence of a consistent beneficial effect.

Several considerations are important when interpreting these broad parent-reported outcomes. First, core symptom-specific measures, including the SNAP-IV inattention subscale and all CPT indices, showed no clear improvement. If the observed CPRS signal mainly reflected improvement in core ADHD symptoms, corresponding improvements in these dedicated core symptom or attention-related measures would be expected; yet, none were observed. Second, both CPRS and CBCL are broad parent-reported instruments that include domains beyond core ADHD symptoms. The CPRS includes dimensions such as oppositional behavior, anxiety, social problems, and psychosomatic complaints [59], whereas the CBCL provides broad assessments of internalizing and externalizing problems [62].

Unfortunately, due to inconsistent reporting of subscale scores across the included studies, a direct meta-analysis of these domain-specific emotional and behavioral subscales was not feasible. Therefore, the small reduction in CPRS total scores should be interpreted as an exploratory signal of possible improvement in broad parent-reported behavioral symptoms, rather than as definitive evidence of improvement in specific emotional regulation, behavioral adaptability, or other clearly defined emotional–behavioral domains. The non-significant CBCL result further weakens the overall evidence for emotional–behavioral benefits. This interpretation is indirect and should not be overstated, because total scores do not identify which specific emotional or behavioral domains may have contributed to the observed CPRS change.

The potential mechanisms underlying these exploratory findings may involve the microbiota–gut–brain axis, a bidirectional communication system connecting the gut microbiota and the central nervous system [9,10,11,63]. Probiotic supplementation may theoretically influence neurotransmitter metabolism, including pathways related to serotonin (5-HT) [15,53,54,55], dopamine (DA) [64,65], and γ-aminobutyric acid (GABA) [55,66,67,68], which are relevant to mood and behavioral regulation. Strains such as *Lactobacillus rhamnosus* and *Bifidobacterium longum* may increase tryptophan availability and serotonin production [69,70], while short-chain fatty acids (SCFAs) may influence dopaminergic and glutamatergic signaling [71,72].

In addition, probiotics may exert anti-inflammatory and immunomodulatory effects by strengthening the intestinal barrier and reducing lipopolysaccharide (LPS) translocation, thereby attenuating systemic and neuroinflammation [56,57,58]. SCFAs such as butyrate may inhibit NF-κB activation and microglial overactivation [73,74], and may promote brain-derived neurotrophic factor (BDNF) expression [75], which is relevant to neuroplasticity. Probiotics may also stabilize the hypothalamic–pituitary–adrenal (HPA) axis, mitigate stress-related reactivity, and support blood–brain barrier integrity [76].

Although these mechanisms provide biological plausibility, they were not directly tested in most of the included ADHD trials. Few studies assessed gut microbiota composition, microbial metabolites, inflammatory markers, neurotransmitter-related metabolites, or other biological mediators. Therefore, the present meta-analysis cannot determine whether the observed CPRS signal was mediated through gut–brain axis pathways. Future trials incorporating microbiome and biomarker assessments are needed to clarify the biological mechanisms underlying any potential emotional–behavioral effects.

### 4.3. Interpretation of SNAP-IV Findings

No significant improvement was observed for SNAP-IV inattention scores, and an apparent paradoxical increase in hyperactivity scores was observed. However, this finding should be interpreted with caution.

The analysis was based on only two studies, with one study contributing almost all of the weight (98.98%), resulting in substantial imbalance [44,46]. This suggests that the observed effect is likely driven by a single dataset rather than representing a consistent pattern across studies.

Taken together, the apparent increase in hyperactivity scores is more likely to reflect statistical instability and study-specific effects rather than a true adverse effect of probiotic supplementation on hyperactivity symptoms.

### 4.4. Comparison with Previous Studies

The findings of the present meta-analysis are consistent with emerging evidence indicating that probiotic supplementation has limited effects on ADHD-related clinical outcomes and specific core symptom-related measures. Recent quantitative syntheses have reported that probiotic supplementation does not significantly reduce overall ADHD severity or core symptom domains, including inattention, hyperactivity, and impulsivity, when compared with placebo, highlighting the limited efficacy of microbiota-based interventions for primary neurocognitive manifestations [30].

Importantly, Allahyari et al. systematically reviewed randomized controlled trials involving probiotic supplementation in children and adolescents with ADHD and concluded that, although these interventions should not replace pharmacological treatments, they may have potential adjunctive value for broader behavioral or emotional outcomes [77]. This partially aligns with our findings. In the present meta-analysis, probiotic supplementation showed a small exploratory reduction in CPRS total scores, but this finding was not consistently supported by CBCL outcomes under the random-effects model. No clear improvements were observed in SNAP-IV inattention scores or CPT-derived attention-related performance measures. Together, these findings suggest that current evidence does not support probiotic supplementation as a direct treatment for core attentional or executive dysfunction, while any potential effect on broader parent-reported behavioral outcomes remains preliminary and uncertain.

Nevertheless, the possibility that earlier or more prolonged supplementation might improve core symptoms should not be dismissed. Several lines of evidence suggest that the timing of intervention is critical. For example, in the Pärtty et al. RCT (2015), early *Lactobacillus rhamnosus GG* supplementation during infancy was associated with a lower childhood ADHD diagnosis rate (*p* = 0.008), and the treatment effect persisted for 13 years [61]. However, this finding reflects early-life preventive supplementation and is not directly comparable with the short-term supplementation trials included in the present meta-analysis, which enrolled children and adolescents with established ADHD diagnoses. Further support comes from longitudinal birth cohort studies. Aatsinki et al. (2019) found that gut microbiota composition in 2.5-month-old infants was associated with temperament traits (e.g., regulatory capacity, orienting duration) at 6 months of age, indicating that early microbial colonization influences fundamental neurobehavioral development [78].

More directly, Brinth et al. (2026) reported in the COPSAC2010 cohort that neonatal gut *Bifidobacterium* levels at week 1 were associated with ADHD risk at age 10, mediated by the tryptophan metabolite indole-3-lactic acid, highlighting the importance of specific colonization patterns during the neonatal window [79]. Taken together, these findings suggest that early-life microbial colonization may be relevant to later neurobehavioral development, but they do not provide direct evidence that short-term probiotic supplementation improves core ADHD symptoms in children who already have a diagnosis.

Several early clinical trials also provide some biological and clinical plausibility for potential behavioral effects. In a randomized, double-blind, placebo-controlled pilot study involving children and adolescents with ADHD and/or ASD, probiotic supplementation was associated with changes in hyperactivity–impulsivity and quality-of-life measures [29]. However, these findings should be interpreted cautiously because pilot trials are typically small and exploratory, and effects observed in mixed neurodevelopmental populations may not directly generalize to children with ADHD alone.

Taken together, the current findings and previous evidence converge in suggesting that probiotic supplementation has not demonstrated a clear benefit for overall ADHD-related clinical outcomes or specific core symptom-related measures in children and adolescents diagnosed with ADHD. A small exploratory signal was observed for CPRS total scores in the present meta-analysis, but this was not consistently supported by CBCL outcomes. Therefore, the potential role of probiotic supplementation in broader emotional–behavioral outcomes remains uncertain. Early-life or preventive supplementation represents a distinct and promising direction for future research, and adequately powered randomized trials with longer follow-up are needed to clarify whether timing, duration, strain specificity, or baseline microbiota profiles modify treatment effects.

### 4.5. Strengths and Limitations

This study has two strengths. First, to our knowledge, this is the first meta-analysis of probiotic supplementation in children and adolescents with ADHD that restricted eligibility to randomized controlled trials enrolling participants diagnosed according to DSM-IV-TR or DSM-5 criteria. This requirement reduced diagnostic heterogeneity and improved the clinical interpretability of the pooled findings [34].

Second, this study addressed an important gap in the existing literature by distinguishing overall ADHD-related clinical outcomes, more specific core symptom-related measures, and broader emotional–behavioral outcomes. Previous evidence has not clearly separated these outcome domains, making it difficult to determine whether probiotic supplementation is more likely to affect core ADHD symptoms, broader parent-reported behavioral outcomes, or neither [35,36,37,38,39].

However, several limitations should be acknowledged. First, the number of included studies was relatively small, and several subgroup analyses included only two or three studies. This may reduce statistical power, limit the precision of pooled estimates, and make subgroup findings sensitive to individual trials [40,80].

Second, most trials had short intervention durations of 8 to 12 weeks and lacked longer-term follow-up. Given that ADHD is a chronic neurodevelopmental condition, short-term supplementation may be insufficient to produce sustained changes in core symptoms, broader emotional–behavioral outcomes, or gut microbiota composition [31].

Third, substantial heterogeneity existed across the included studies. Trials differed in probiotic strains, multistrain formulations, daily CFU doses, intervention duration, comparator conditions, background treatment, and outcome measures. Because probiotic effects may be strain-specific and dose-dependent, the current evidence does not allow firm conclusions regarding the optimal strain, dose, formulation, or treatment duration [23,24,81].

Fourth, comparator conditions were inconsistent across trials. Some studies used placebo controls, whereas others used standard treatment or medication-only control condition [27,28,29,41,42,43,44,45,46]. This variation complicates the interpretation of pooled effects because the observed differences may reflect not only the effect of probiotic supplementation itself, but also differences in background treatment or comparator design.

Fifth, concomitant medication use was frequent and inconsistent across the included trials. Several studies evaluated probiotic supplementation as an adjunct to methylphenidate, atomoxetine, or other psychotropic medications, whereas others included participants who were not receiving pharmacological treatment [27,28,41,43,44,46]. Concomitant pharmacotherapy may substantially influence baseline symptom severity, treatment responsiveness, parent-reported behavioral ratings, and the magnitude of any additional effect attributable to probiotic supplementation. Therefore, differences in medication status and background treatment may represent an important source of clinical heterogeneity. Because the number of included studies was small and medication status, medication dose, and treatment stability were not consistently reported across trials, subgroup analyses according to medication use were not feasible. This limitation should be considered when interpreting the pooled estimates and the exploratory CPRS finding.

Sixth, the included studies enrolled participants across a relatively wide age range, approximately from 4 to 18 years. Age may act as an important effect modifier because gut microbiota composition, neurodevelopmental stage, emotional and behavioral regulation, ADHD symptom presentation, medication responsiveness, and parent-reported behavioral ratings can differ substantially between preschool children, school-aged children, and adolescents. Therefore, pooling studies across this age range may have introduced additional clinical heterogeneity and may have obscured age-specific effects of probiotic supplementation. Because the number of included trials was limited and age-stratified outcome data were generally unavailable, subgroup analyses by developmental stage could not be performed.

Seventh, outcome measures varied substantially across studies, and the broad exploratory synthesis combined instruments that assessed related but not identical constructs. Although SMDs improved statistical comparability across different scales, they did not eliminate conceptual heterogeneity among ADHD-specific symptom scales, broad parent-reported behavioral scales, and CPT-derived performance measures. Therefore, the overall pooled estimate should not be interpreted as a construct-specific estimate of core ADHD symptom improvement. Domain-specific analyses were considered more clinically interpretable, but these analyses were limited by the small number of studies available for each outcome. In addition, CPT-derived indices have limitations in ecological validity. Although CPTs provide standardized laboratory-based measures of attention-related performance, they do not necessarily reflect day-to-day ADHD-related impairment, academic functioning, social functioning, or overall clinical improvement. Therefore, the CPT findings should be interpreted as performance-based attention measures rather than direct evidence of objective clinical change.

Eighth, reliance on parent-reported measures may introduce reporting bias. Parent ratings are clinically meaningful and commonly used in pediatric ADHD research, but they may be influenced by parental expectations, awareness of treatment allocation, perceived changes in general behavior, or changes in family context [82]. This limitation is particularly relevant to broad behavioral scales and should be considered when interpreting the exploratory CPRS finding and the non-significant finding for CBCL total scores.

Ninth, the clinical relevance of the observed findings remains uncertain. Although CPRS total scores showed a small statistically significant reduction, the effect on CBCL total scores was not statistically significant under the random-effects model. The CPRS effect was small and based on a limited number of heterogeneous trials. It remains unclear whether this finding corresponds to meaningful improvements in daily functioning, academic performance, family interactions, social functioning, quality of life, or long-term prognosis [83]. Therefore, statistical significance should not be equated with clinically meaningful benefit.

Tenth, some outcome data were derived from graphical extraction or statistical estimation, which may introduce measurement uncertainty. In particular, the SNAP-18 data from Skott et al. were converted from item-level mean scores with IQRs to estimated total-score means and SDs using the approximation SD ≈ IQR/1.35. This approach assumes approximate symmetry of the underlying distribution and may be less robust for bounded ordinal symptom scores. Although such estimation methods are commonly used when original numerical data are unavailable, these results should be interpreted cautiously [40].

Finally, most included studies did not assess biological mediators such as gut microbiota composition, microbial metabolites, inflammatory markers, neurotransmitter-related metabolites, or other gut–brain axis biomarkers. This limits mechanistic interpretation and prevents determination of whether the observed CPRS signal or any potential behavioral changes were mediated by microbiota-related pathways [9,10,11,12,13,30]. Moreover, probiotic-induced changes in the gut microbiota may be transient. Evidence from psychiatric populations suggests that probiotic supplementation does not consistently produce significant or sustained alterations in gut microbiota diversity or composition. For example, Ng et al. reported that probiotic supplementation in patients with major depressive disorder produced only modest clinical effects and showed no consistent effects on gut microbiota diversity; in most trials, no significant alterations in gut microbiota composition were observed after four to eight weeks of intervention [31]. Therefore, even if a short-term signal in CPRS total scores is observed, its durability, clinical relevance, and biological significance remain uncertain. Taken together, these limitations reduce the certainty of the current evidence and preclude firm conclusions regarding the clinical efficacy, optimal formulation, durability, and biological mechanisms of probiotic supplementation in children and adolescents with ADHD.

### 4.6. Clinical and Research Implications

From a clinical standpoint, current randomized controlled trial evidence does not support the use of probiotic supplementation as an established standalone treatment for overall ADHD-related clinical outcomes or specific core symptom-related measures in children and adolescents. A small exploratory reduction in CPRS total scores may suggest possible improvement in some parent-reported behavioral outcomes; however, this finding was not consistently supported by CBCL outcomes under the random-effects model and should therefore be interpreted cautiously. Given its potential acceptability and microbiota-targeted rationale, probiotic supplementation may still be considered a promising adjunctive strategy for further investigation, but current evidence is insufficient to support firm clinical recommendations [77].

From a research perspective, future randomized controlled trials should employ standardized probiotic formulations, clearly defined strains and doses, appropriate placebo controls, longer intervention and follow-up periods, and adequately powered sample sizes. Overall ADHD-related clinical outcomes, specific core symptom-related measures, and broader emotional–behavioral outcomes should be reported separately, and outcome selection should be predefined to reduce selective reporting. In addition, future studies should incorporate multimodal outcomes, including behavioral ratings, objective cognitive measures, gut microbiota composition, microbial metabolites, inflammatory markers, and other gut–brain axis-related biomarkers. Such studies will help clarify whether probiotic supplementation produces sustained biological changes and whether these changes are associated with clinically meaningful benefits in children and adolescents with ADHD [84].

## 5. Conclusions

In conclusion, current 8–12-week RCT evidence does not demonstrate a clear benefit of probiotic supplementation for ADHD core symptom-related measures in children and adolescents with ADHD, including SNAP-IV inattention scores and CPT-derived attention-related performance measures. However, exploratory findings suggest that probiotic supplementation may have potential for improving broad parent-reported behavioral and emotional difficulties, particularly CPRS-assessed behavioral symptoms. This potential benefit should be interpreted cautiously because it was based on a small number of heterogeneous trials and was not consistently supported by CBCL outcomes under the random-effects model. Although probiotic supplementation may represent a potential adjunctive strategy for broad parent-reported behavioral and emotional difficulties in children and adolescents with ADHD, current evidence remains insufficient to support firm clinical recommendations. Larger, rigorously designed randomized controlled trials with standardized probiotic formulations, clearly defined strains and doses, longer intervention and follow-up periods, and integrated microbiome and biomarker assessments are needed to confirm these findings and clarify their clinical relevance and underlying mechanisms.

## Figures and Tables

**Table 1:** Summary of characteristics of studies in the current meta-analysis.

Author(s)	Diagnostic (Criteria)	Design	Intervention Group	Control Group	*N*	Duration (Weeks)	Dose (CFU/Day)	Outcome	Medications	Participant Age	Country
Elhossiny et al., 2023 [27]	DSM-5	RCT	Probiotics *Lactobacillus plantarum* LB plus atomoxetine	Atomoxetine only	76	12	1 × 10^10^	CPRS-R:L CPT	100% atomoxetine	6–16	Cairo Egypt
Sangsefidi et al., 2024 [28]	DSM-5	RCT	Probiotics with *Lactobacillus plantarum* PTCC 1896™ (A7) and *Bifidobacterium animalis* subsp. lactis (BB-12^®^).	Placebo	60	8	10^6^–10^7^	CPRS	100% methylphenidate	4–16	Iran
Rojo-Marticella et al., 2024 [29] ^a^	DSM-5	RCT	Probiotics *Lactiplantibacillus* mixture	Placebo	38	12	1 × 10^9^	CPT	Not applicable	5–16	Spain
Ghanaatgar et al., 2022 [41]	DSM-5	RCT	Probiotics mixture (14 strains)	Placebo plus microcrystal cellulose	34	8	2 × 10^9^	CPRS-R:S	100% methylphenidate	6–12	Tehran Iran
Kumperscak et al., 2020 [42]	DSM-5	RCT	Probiotic *Lactobacillus rhamnosus* GG	Placebo without LGG	32	12	≥1 × 10^10^	CBCL	Not applicable	4–17	Maribor Slovenia
Sepehrmanesh et al., 2021 [43]	DSM-IV-TR	RCT	Probiotic	Placebo	34	8	8 × 10^9^	ADHD-RS	100% methylphenidate	8–12	Tehran Iran
Skott et al., 2020 [44]	DSM-V	RCT	Synbiotic 2000	Placebo (maltodextrin)	68	9	4 × 10^11^	CPRS-R:S SNAP-IV	58.8% on different psychotropics (30% on methylphenidate)	5–18	Sweden
Trezzi et al., 2025 [45]	DSM-5	RCT	Synbiotic mix enriched with a pigmented corn extract	Placebo	38	12	3 × 10^9^	CPRS CBCL	Not applicable	6–16	Italy
Wang et al., 2024 [46]	DSM-5	RCT	Probiotics *Bifidobacterium bifidum* Bf-688 plus methylphenidate	Placebo plus methylphenidate	94	12	5 × 10^9^	SNAP-IV CPT	100% methylphenidate	6–12	Taiwan China

ᵃ Rojo-Marticella et al. enrolled children and adolescents with ASD and/or ADHD. In the present review, only ADHD-specific data were extracted and included in the meta-analysis.

## Data Availability

The data presented in this study are available upon request from the corresponding author.

## Supplementary Materials

The following supporting information can be downloaded at: https://www.mdpi.com/article/10.3390/nu18142357/s1, File S1: Search Strategy; File S2: Reasons for Excluded Records; File S3: Rationale for Selection; File S4: Additional Reference Details; File S5: GRADE; File S6: Forest and Funnel Plot (Figure S1: Sensitivity analysis for overall ADHD-related clinical outcomes: using the leave-one-out method; Figure S2: Funnel plot for the analysis of overall ADHD-related clinical outcomes; Figure S3: Forest plot of subgroup analysis for total scores on the CBCL; Figure S4: Forest plot of the SNAP-IV Inattention subgroup analysis; Figure S5: Forest plot of the subgroup analysis for SNAP-IV hyperactivity; Figure S6: Forest plot for subgroup analysis of CPT commission errors; Figure S7: Forest plot for the subgroup analysis of CPT omission errors; Figure S8: Forest plot for the subgroup analysis of CPT detectability); File S7: PRISMA_2020_abstract_checklist; File S8: PRISMA_2020_checklist.

## Abbreviations

The following abbreviations are used in this manuscript:

ADHD	Attention-Deficit/Hyperactivity Disorder
CBCL	Child Behavior Checklist
CENTRAL	Cochrane Central Register of Controlled Trials
CI	Confidence Interval
CPRS	Conners’ Parent Rating Scale
CPRS-R:L	Conners’ Parent Rating Scale-Revised: Long Version
CPRS-R:S	Conners’ Parent Rating Scale-Revised: Short Version
CPT	Continuous Performance Test
DSM-5	Diagnostic and Statistical Manual of Mental Disorders, 5th Edition
GABA	Gamma-Aminobutyric Acid
HPA axis	Hypothalamic–Pituitary–Adrenal Axis
LPS	Lipopolysaccharide
MD	Mean Difference
MeSH	Medical Subject Headings
PRISMA	Preferred Reporting Items for Systematic Reviews and Meta-Analyses
PROSPERO	International Prospective Register of Systematic Reviews
RCT	Randomized Controlled Trial
RoB 2.0	Cochrane Risk of Bias 2.0 Tool
SCFA	Short-Chain Fatty Acid
SMD	Standardized Mean Difference
SNAP-IV	Swanson, Nolan and Pelham—IV Rating Scales
