# Supplementary material for: Probiotic Supplementation in Children and Adolescents with ADHD: A Systematic Review and Meta-Analysis of ADHD-Related and Emotional–Behavioral Outcomes
Source: Nutrients. 2026 Jul 17;18(14):2357. doi: 10.3390/nu18142357 (PMC13415223; doi:10.3390/nu18142357)
Supplement: Supplementary file 1 [file nutrients-18-02357-s001.zip › Supplementary File S1 Search Strategy.pdf]

PubMed

Search: ((((((((((((((((((((((((("Attention Deficit Disorder with Hyperactivity/blood"[Mesh] OR "Attention Deficit Disorder with Hyperactivity/cerebrospinal fluid"[Mesh] OR "Attention Deficit Disorder with Hyperactivity/chemically induced"[Mesh] OR "Attention Deficit Disorder with Hyperactivity/classification"[Mesh] OR "Attention Deficit Disorder with Hyperactivity/complications"[Mesh] OR "Attention Deficit Disorder with Hyperactivity/diagnosis"[Mesh] OR "Attention Deficit Disorder with Hyperactivity/diagnostic imaging"[Mesh] OR "Attention Deficit Disorder with Hyperactivity/diet therapy"[Mesh] OR "Attention Deficit Disorder with Hyperactivity/drug therapy"[Mesh] OR "Attention Deficit Disorder with Hyperactivity/economics"[Mesh] OR "Attention Deficit Disorder with Hyperactivity/enzymology"[Mesh] OR "Attention Deficit Disorder with Hyperactivity/epidemiology"[Mesh] OR "Attention Deficit Disorder with Hyperactivity/ethnology"[Mesh] OR "Attention Deficit Disorder with Hyperactivity/etiology"[Mesh] OR "Attention Deficit Disorder with Hyperactivity/genetics"[Mesh] OR "Attention Deficit Disorder with Hyperactivity/history"[Mesh] OR "Attention Deficit Disorder with Hyperactivity/immunology"[Mesh] OR "Attention Deficit Disorder with Hyperactivity/metabolism"[Mesh] OR "Attention Deficit Disorder with Hyperactivity/microbiology"[Mesh] OR "Attention Deficit Disorder with Hyperactivity/mortality"[Mesh] OR "Attention Deficit Disorder with Hyperactivity/nursing"[Mesh] OR "Attention Deficit Disorder with Hyperactivity/parasitology"[Mesh] OR "Attention Deficit Disorder with Hyperactivity/pathology"[Mesh] OR "Attention Deficit Disorder with Hyperactivity/physiopathology"[Mesh] OR "Attention Deficit Disorder with Hyperactivity/prevention and control"[Mesh] OR "Attention Deficit Disorder with Hyperactivity/psychology"[Mesh] OR "Attention Deficit Disorder with Hyperactivity/rehabilitation"[Mesh] OR "Attention Deficit Disorder with Hyperactivity/surgery"[Mesh] OR "Attention Deficit Disorder with Hyperactivity/therapy"[Mesh] OR "Attention Deficit Disorder with Hyperactivity/urine"[Mesh] OR "Attention Deficit Disorder with Hyperactivity/virology"[Mesh])) OR (ADHD[Title/Abstract])) OR (ADDH[Title/Abstract])) OR (Attention Deficit Disorders with Hyperactivity[Title/Abstract])) OR (Attention Deficit

Hyperactivity Disorders[Title/Abstract])) OR (Attention Deficit Hyperactivity Disorder[Title/Abstract])) OR (Attention Deficit-Hyperactivity Disorder[Title/Abstract])) OR (Attention Deficit-Hyperactivity Disorders[Title/Abstract])) OR (Deficit-Hyperactivity Disorder, Attention[Title/Abstract])) OR (Deficit-Hyperactivity Disorders, Attention[Title/Abstract])) OR (Disorder, Attention Deficit-Hyperactivity[Title/Abstract])) OR (Disorders, Attention Deficit-Hyperactivity[Title/Abstract])) OR (Hyperkinetic Syndrome[Title/Abstract])) OR (Syndromes, Hyperkinetic[Title/Abstract])) OR (Attention Deficit Disorder[Title/Abstract])) OR (Attention Deficit Disorders[Title/Abstract])) OR (Deficit Disorder, Attention[Title/Abstract])) OR (Deficit Disorders, Attention[Title/Abstract])) OR (Disorder, Attention Deficit[Title/Abstract])) OR (Disorders, Attention Deficit[Title/Abstract])) OR (Brain Dysfunction, Minimal[Title/Abstract])) OR (Dysfunction, Minimal Brain[Title/Abstract])) OR (Minimal Brain Dysfunction[Title/Abstract])) AND ((((((((((((((((((("Adolescent/legislation and jurisprudence"[Mesh] OR "Adolescent/physiology"[Mesh])) OR (Adolescents[Title/Abstract])) OR (Adolescence[Title/Abstract])) OR (Adolescents, Female[Title/Abstract])) OR (Adolescent, Female[Title/Abstract])) OR (Female Adolescent[Title/Abstract])) OR (Female Adolescents[Title/Abstract])) OR (Adolescents, Male[Title/Abstract])) OR (Adolescent, Male[Title/Abstract])) OR (Male Adolescent[Title/Abstract])) OR (Male Adolescents[Title/Abstract])) OR (Youth[Title/Abstract])) OR (Youths[Title/Abstract])) OR (Teens[Title/Abstract])) OR (Teen[Title/Abstract])) OR (Teenagers[Title/Abstract])) OR (Teenager[Title/Abstract])) OR (Children[Title/Abstract])) OR (Child[Title/Abstract])) OR (Minors[Title/Abstract]))) AND ("probiotics/administration and dosage"[MeSH Terms] OR "probiotics/adverse effects"[MeSH Terms] OR "probiotics/analysis"[MeSH Terms] OR "probiotics/chemical synthesis"[MeSH Terms] OR "probiotics/chemistry"[MeSH Terms] OR "probiotics/classification"[MeSH Terms] OR "probiotics/economics"[MeSH Terms] OR "probiotics/history"[MeSH Terms] OR "probiotics/isolation and purification"[MeSH Terms] OR "probiotics/metabolism"[MeSH Terms] OR "probiotics/pharmacokinetics"[MeSH Terms] OR "probiotics/pharmacology"[MeSH Terms] OR "probiotics/poisoning"[MeSH Terms] OR "probiotics/radiation effects"[MeSH Terms] OR "probiotics/standards"[MeSH Terms] OR "probiotics/supply and distribution"[MeSH Terms] OR "probiotics/therapeutic use"[MeSH Terms] OR "probiotics/toxicity"[MeSH Terms] OR "Probiotic"[Title/Abstract] OR

"synbiotics/administration and dosage"[MeSH Terms] OR "synbiotics/adverse effects"[MeSH Terms] OR "synbiotics/analysis"[MeSH Terms] OR "synbiotics/classification"[MeSH Terms] OR "synbiotics/economics"[MeSH Terms] OR "synbiotics/standards"[MeSH Terms] OR "synbiotics/statistics and numerical data"[MeSH Terms] OR "Synbiotic"[Title/Abstract] OR "prebiotics/administration and dosage"[MeSH Terms] OR "prebiotics/adverse effects"[MeSH Terms] OR "prebiotics/analysis"[MeSH Terms] OR "prebiotics/classification"[MeSH Terms] OR "prebiotics/economics"[MeSH Terms] OR "prebiotics/history"[MeSH Terms] OR "prebiotics/microbiology"[MeSH Terms] OR "prebiotics/parasitology"[MeSH Terms] OR "prebiotics/standards"[MeSH Terms] OR "prebiotics/statistics and numerical data"[MeSH Terms] OR "prebiotics/supply and distribution"[MeSH Terms] OR "prebiotics/toxicity"[MeSH Terms] OR "Prebiotic"[Title/Abstract])

Embase

('attention'/exp OR attention) AND deficit AND ('disorder'/exp OR disorder) AND with AND ('hyperactivity'/exp OR hyperactivity) OR 'attention deficit disorders with hyperactivity':ab,ti OR 'addh':ab,ti OR 'adhd':ab,ti OR 'attention deficit hyperactivity disorders':ab,ti OR 'attention deficit hyperactivity disorder':ab,ti OR 'attention deficit-hyperactivity disorder':ab,ti OR 'attention deficit-hyperactivity disorders':ab,ti OR 'deficit-hyperactivity disorder, attention':ab,ti OR 'deficit-hyperactivity disorders, attention':ab,ti OR 'disorder, attention deficit-hyperactivity':ab,ti OR 'disorders, attention deficit-hyperactivity':ab,ti OR 'hyperkinetic syndrome':ab,ti OR 'syndromes, hyperkinetic':ab,ti OR 'attention deficit disorder':ab,ti OR 'attention deficit disorders':ab,ti OR 'deficit disorder, attention':ab,ti OR 'deficit disorders, attention':ab,ti OR 'disorder, attention deficit':ab,ti 'disorders, attention deficit':ab,ti OR 'brain dysfunction, minimal':ab,ti OR 'dysfunction, minimal brain':ab,ti 'minimal brain dysfunction':ab,ti

AND

adolescent OR child OR 'adolescents':ab,ti OR 'adolescence':ab,ti OR 'adolescents, female':ab,ti OR 'adolescent, female':ab,ti OR 'female, adolescent':ab,ti OR 'female adolescents':ab,ti OR 'adolescents, male':ab,ti OR 'adolescent, male':ab,ti OR 'male, adolescent':ab,ti OR 'male adolescents':ab,ti OR 'youth':ab,ti OR 'youths':ab,ti OR 'teens':ab,ti OR 'teen':ab,ti OR 'teenagers':ab,ti OR 'teenager':ab,ti OR 'children':ab,ti

AND

probiotics OR 'probiotic':ab,ti OR synbiotics OR 'synbiotic':ab,ti OR prebiotics OR 'prebiotic':ab,ti

The Cochrane Central Register of Controlled Trials (CENTRAL)

MeSH descriptor: [Attention Deficit Disorder with Hyperactivity] explode all trees

OR

(ADHD):ab,ti,kw OR (ADDH):ab,ti,kw OR (Attention Deficit Disorders with Hyperactivity):ab,ti,kw OR (Attention Deficit Hyperactivity Disorders):ab,ti,kw OR (Attention Deficit Hyperactivity Disorder):ab,ti,kw OR (Attention Deficit-Hyperactivity Disorder):ab,ti,kw OR (Attention Deficit-Hyperactivity Disorders):ab,ti,kw OR (Deficit-Hyperactivity Disorder, Attention):ab,ti,kw OR (Deficit-Hyperactivity Disorders, Attention):ab,ti,kw OR (Disorder, Attention Deficit-Hyperactivity):ab,ti,kw OR (Disorders, Attention Deficit-Hyperactivity):ab,ti,kw OR (Hyperkinetic Syndrome):ab,ti,kw OR (Syndromes, Hyperkinetic):ab,ti,kw OR (Attention Deficit Disorder):ab,ti,kw OR (Attention Deficit Disorders):ab,ti,kw OR (Deficit Disorder, Attention):ab,ti,kw OR (Deficit Disorders, Attention):ab,ti,kw OR (Disorder, Attention Deficit):ab,ti,kw OR (Disorders, Attention Deficit):ab,ti,kw OR (Brain Dysfunction, Minimal):ab,ti,kw OR (Dysfunction, Minimal Brain):ab,ti,kw OR (Minimal Brain Dysfunction):ab,ti,kw

AND

MeSH descriptor: [Synbiotics] explode all trees

OR

(synbiotic):ab,ti,kw

OR

MeSH descriptor: [Probiotics] explode all trees

OR

(probiotic):ab,ti,kw

OR

MeSH descriptor: [Prebiotics] explode all trees

OR

(prebiotic):ab,ti,kw

AND

MeSH descriptor: [Adolescent] explode all trees

OR

(Adolescents):ab,ti,kw OR (Adolescence):ab,ti,kw OR (Adolescents, Female):ab,ti,kw OR (Adolescent, Female):ab,ti,kw OR (Female Adolescent):ab,ti,kw OR (Female Adolescents):ab,ti,kw OR (Adolescents, Male):ab,ti,kw OR (Adolescent, Male):ab,ti,kw OR (Male Adolescent):ab,ti,kw OR (Male Adolescents):ab,ti,kw OR (Youth):ab,ti,kw OR (Youths):ab,ti,kw OR (Teens):ab,ti,kw OR (Teen):ab,ti,kw OR (Teenagers):ab,ti,kw OR (Teenager):ab,ti,kw OR (Minor):ab,ti,kw OR(Minors):ab,ti,kw

OR

MeSH descriptor: [Child] explode all trees

OR

(Children):ab,ti,kw

PsycINFO

(su.exact("Adolescents") OR ti("Adolescent" OR "Adolescence" OR "Adolescents, Female" OR "Adolescent, Female" OR "Female Adolescent" OR "Female Adolescents" OR "Adolescents, Male" OR "Adolescent, Male" OR "Male Adolescent" OR "Male Adolescents" OR "Youth" OR "Youths" OR "Teens" OR "Teen" OR "Teenagers" OR "Teenager" OR "Children" OR "Child") OR ab("Adolescent" OR "Adolescence" OR "Adolescents, Female" OR "Adolescent, Female" OR "Female Adolescent" OR "Female Adolescents" OR "Adolescents, Male" OR "Adolescent, Male" OR "Male Adolescent" OR "Male Adolescents" OR "Youth" OR "Youths" OR "Teens" OR "Teen" OR "Teenagers" OR "Teenager" OR "Children" OR "Child"))

AND

(su.exact("Attention Deficit Disorder with Hyperactivity") OR ti("ADHD" OR "ADDH" OR "Attention Deficit Disorders with Hyperactivity" OR "Attention Deficit Hyperactivity Disorders" OR "Attention Deficit Hyperactivity Disorder" OR "Attention Deficit-Hyperactivity Disorder" OR "Attention Deficit-Hyperactivity Disorders" OR "Deficit-Hyperactivity Disorder, Attention" OR "Deficit-Hyperactivity Disorders, Attention" OR "Disorder, Attention Deficit-Hyperactivity" OR "Disorders, Attention Deficit-Hyperactivity" OR "Hyperkinetic Syndrome" OR "Syndromes, Hyperkinetic" OR "Attention Deficit Disorder" OR "Attention Deficit Disorders" OR "Deficit Disorder, Attention" OR "Deficit Disorders, Attention" OR "Disorder, Attention Deficit" OR "Disorders, Attention Deficit" OR "Brain Dysfunction, Minimal" OR "Dysfunction, Minimal Brain" OR "Minimal Brain Dysfunction") OR ab("ADHD" OR "ADDH" OR "Attention Deficit Disorders with Hyperactivity" OR "Attention Deficit Hyperactivity Disorders" OR "Attention Deficit Hyperactivity Disorder" OR "Attention Deficit-Hyperactivity Disorder" OR "Attention Deficit-Hyperactivity Disorders" OR "Deficit-Hyperactivity Disorder, Attention" OR "Deficit-Hyperactivity Disorders, Attention" OR "Disorder, Attention Deficit-Hyperactivity" OR "Disorders, Attention Deficit-Hyperactivity" OR "Hyperkinetic Syndrome" OR "Syndromes, Hyperkinetic" OR "Attention Deficit Disorder" OR "Attention Deficit Disorders" OR "Deficit Disorder, Attention" OR "Deficit Disorders, Attention" OR "Disorder, Attention Deficit" OR "Disorders, Attention Deficit" OR "Brain Dysfunction, Minimal" OR "Dysfunction, Minimal Brain" OR "Minimal Brain Dysfunction"))

AND

(su.exact("synbiotics" OR "prebiotics" OR "probiotics") OR ti("synbiotics" OR "prebiotics" OR "probiotics" OR "probiotic therapy" OR "probiotic supplement") OR ab("synbiotics" OR "prebiotics" OR "probiotics" OR "probiotic therapy" OR "probiotic supplement"))

EBSCO

Query: (SU adolescents OR TI (Adolescent OR Adolescence OR Adolescents, Female OR Adolescent, Female OR Female Adolescent OR Female Adolescents OR Adolescents, Male OR Adolescent, Male OR Male Adolescent OR Male Adolescents OR Youth OR Youths OR Teens OR Teen OR Teenagers OR Teenager) OR AB (Adolescent OR Adolescence OR Adolescents, Female OR Adolescent, Female OR Female Adolescent OR Female Adolescents OR Adolescents, Male OR Adolescent, Male OR Male Adolescent OR Male Adolescents OR Youth OR Youths OR Teens OR Teen OR Teenagers OR Teenager OR Children OR Child)) AND (SU attention deficit disorder with hyperactivity OR TI (ADHD OR ADDH OR Attention Deficit Disorders with Hyperactivity OR Attention Deficit Hyperactivity Disorders OR Attention Deficit Hyperactivity Disorder OR Attention Deficit-Hyperactivity Disorder OR Attention Deficit-Hyperactivity Disorders OR Deficit-Hyperactivity Disorder, Attention OR Deficit-Hyperactivity Disorders, Attention OR Disorder, Attention Deficit-Hyperactivity OR Disorders, Attention Deficit-Hyperactivity OR Hyperkinetic Syndrome OR Syndromes, Hyperkinetic OR Attention Deficit Disorder OR Attention Deficit Disorders OR Deficit Disorder, Attention OR Deficit Disorders, Attention OR Disorder, Attention Deficit OR Disorders, Attention Deficit OR Brain Dysfunction, Minimal OR Dysfunction, Minimal Brain OR Minimal Brain Dysfunction) OR AB (ADHD OR ADDH OR Attention Deficit Disorders with Hyperactivity OR Attention Deficit Hyperactivity Disorders OR Attention Deficit Hyperactivity Disorder OR Attention Deficit-Hyperactivity Disorder OR Attention Deficit-Hyperactivity Disorders OR Deficit-Hyperactivity Disorder, Attention OR Deficit-Hyperactivity Disorders, Attention OR Disorder, Attention Deficit-Hyperactivity OR Disorders, Attention Deficit-Hyperactivity OR Hyperkinetic Syndrome OR Syndromes, Hyperkinetic OR Attention Deficit Disorder OR Attention Deficit Disorders OR Deficit Disorder, Attention OR Deficit Disorders, Attention OR Disorder, Attention Deficit OR Disorders, Attention Deficit OR Brain Dysfunction, Minimal OR Dysfunction, Minimal Brain OR Minimal Brain Dysfunction)) AND ((SU synbiotics OR TI synbiotics OR AB synbiotics) OR (SU prebiotics OR TI prebiotics OR AB prebiotics) OR (SU probiotics OR TI (probiotics or probiotic therapy or probiotic supplement) OR AB (probiotics or probiotic therapy or probiotic supplement)))
