# Supplementary material for: Probiotic Supplementation in Children and Adolescents with ADHD: A Systematic Review and Meta-Analysis of ADHD-Related and Emotional–Behavioral Outcomes
Source: Nutrients. 2026 Jul 17;18(14):2357. doi: 10.3390/nu18142357 (PMC13415223; doi:10.3390/nu18142357)
Supplement: Supplementary file 1 [file nutrients-18-02357-s001.zip › Supplementary File S3 Rationale for selection.pdf]

No.	Study	Assessment Tool	Rationale for Selection
1	Sangsefidi et al. 2024	CPRS (Conners Parent Rating Scale)	A comprehensive parent-reported scale covering multiple psychiatric and behavioral comorbidity dimensions in addition to ADHD core symptoms.
2	Rojo-Marticella et al. 2024	CPT (Omission Errors)	Since no total score of other comprehensive scales was provided, omission errors were extracted as they represent an objective cognitive marker of sustained attention deficits, directly reflecting the core attention impairment dimension of ADHD.
3	Wang et al. 2024	SNAP-IV (Inattention)	Because no total score of other comprehensive scales was available, the SNAP-IV Inattention subscale was selected, as it is the core assessment indicator for "ADHD predominantly inattentive type".
4	Elhossiny et al. 2023	CPRS-R-L (Conners Parent Rating Scale-Revised Long Form)	A comprehensive parent-reported scale covering multiple psychiatric and behavioral comorbidity dimensions in addition to ADHD core symptoms.
5	Sepehrmanesh et al. 2021	ADHD-RS (ADHD Rating Scale)	A specialized clinical scale specifically designed to assess ADHD core symptoms (inattention and hyperactivity/impulsivity).
6	Kumperscak et al. 2020	CBCL (Achenbach Child Behavior Checklist)	An internationally recognized comprehensive behavioral scale encompassing a wide range of internalizing (e.g., anxiety, depression) and externalizing (e.g., aggression, rule-breaking) problems.

7	Trezzi et al. 2023	CPRS (Conners Parent Rating Scale)	A comprehensive parent-reported scale covering multiple psychiatric and behavioral comorbidity dimensions in addition to ADHD core symptoms.
8	Skott et al. 2020	SNAP-IV (Inattention)	As no total score of other comprehensive scales was available, the SNAP-IV Inattention subscale was selected, as it is the core assessment indicator for "ADHD predominantly inattentive type".
9	Ghanasatgar et al. 2022	CPRS-RS (Conners Parent Rating Scale-Revised Short Form)	A short-form version of the CPRS, which is easy to administer and focuses on identifying a wide range of behavioral manifestations of ADHD.
