# Supplementary material for: Probiotic Supplementation in Children and Adolescents with ADHD: A Systematic Review and Meta-Analysis of ADHD-Related and Emotional–Behavioral Outcomes
Source: Nutrients. 2026 Jul 17;18(14):2357. doi: 10.3390/nu18142357 (PMC13415223; doi:10.3390/nu18142357)
Supplement: Supplementary file 1 [file nutrients-18-02357-s001.zip › Supplementary File S5 GRADE.pdf]

Certainty assessment							№ of patients		Effect		Certainty	Importance
№ of studies	Study design	Risk of bias	Inconsistency	Indirectness	Imprecision	Other considerations	Probiotic supplementation	comparison	Relative (95% CI)	Absolute (95% CI)		
**Overall ADHD – related clinical outcomes (follow-up: range 8 weeks to 12 weeks; assessed using ADHD-RS, CPRS, CBCL, SNAP-IV, and CPT-derived indices; pooled using SMD))**												
9	randomised trials	serious	not serious	serious[a]	serious[b]	none[c]	252	230	-	SMD **0.25 SD lower** (0.57 lower to 0.07 higher)	⨁◯◯◯ Very low[a,b,c]	

CI: confidence interval; **SMD:** standardised mean difference

Explanations

a. We downgraded by one level for indirectness because the pooled outcome combined data from multiple different assessment instruments (including CPRS, SNAP, ADHD-RS, CPT, and CBCL) to evaluate ADHD-related symptoms and functional domains. The use of these different tools introduces methodological variability that limits the direct comparability of the constructs being measured; thus, we conservatively downgraded for indirectness. Moreover, substantial heterogeneity further warranted a downgrade for inconsistency. Accordingly, the overall confidence in this pooled estimate is rated as very low.
b. We downgraded the certainty of evidence by one level for serious imprecision. Although the total sample size (N = 482) exceeded the optimal information size (OIS) threshold of 400 for continuous outcomes, the 95% confidence interval of the pooled effect estimate (SMD -0.25, 95% CI -0.57 to 0.07) crossed the null value (0). Moreover, the overall effect was not statistically significant (P = 0.13). This indicates that the current evidence cannot rule out the possibility of no effect, and the precision of the effect estimate is insufficient to draw a firm conclusion.
c. We did not downgrade for publication bias. Visual inspection of the funnel plot showed no obvious asymmetry. Additionally, we conducted a comprehensive search across multiple databases (including clinical trial registries) and included both published and unpublished studies if available. Although the number of included studies is less than 10, which limits the power of formal tests (e.g., Egger's test), the visual assessment suggests a low probability of publication bias
