# Supplementary material for: Probiotic Supplementation in Children and Adolescents with ADHD: A Systematic Review and Meta-Analysis of ADHD-Related and Emotional–Behavioral Outcomes
Source: Nutrients. 2026 Jul 17;18(14):2357. doi: 10.3390/nu18142357 (PMC13415223; doi:10.3390/nu18142357)
Supplement: Supplementary file 1 [file nutrients-18-02357-s001.zip › Supplementary File S6 Forest and Funnel Plot.pdf]

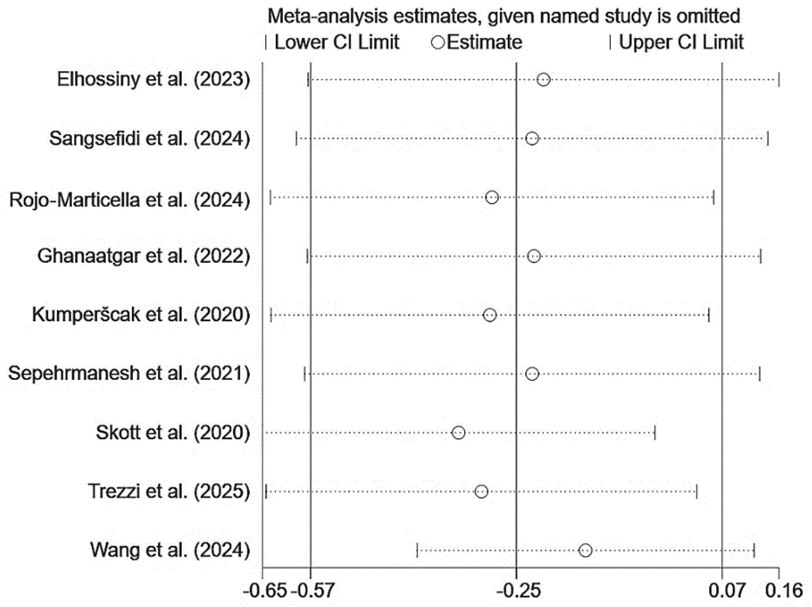

Figure S1. Sensitivity analysis for overall ADHD-related clinical outcomes: using the leave-one-out method.

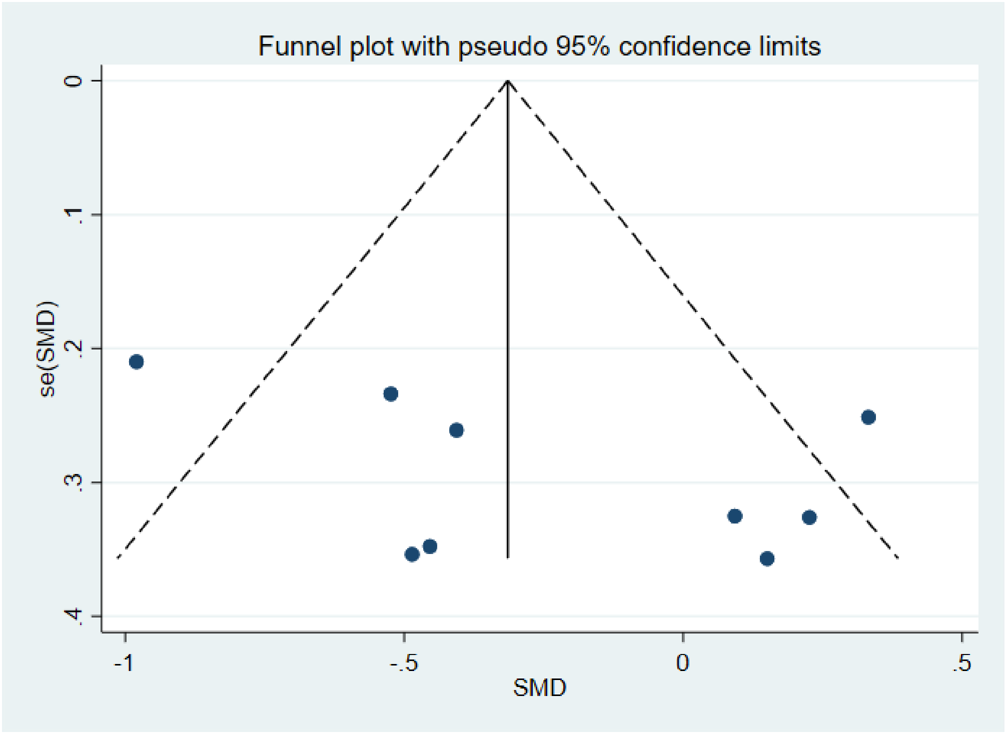

Figure S2. Funnel plot for the analysis of overall ADHD-related clinical outcomes.

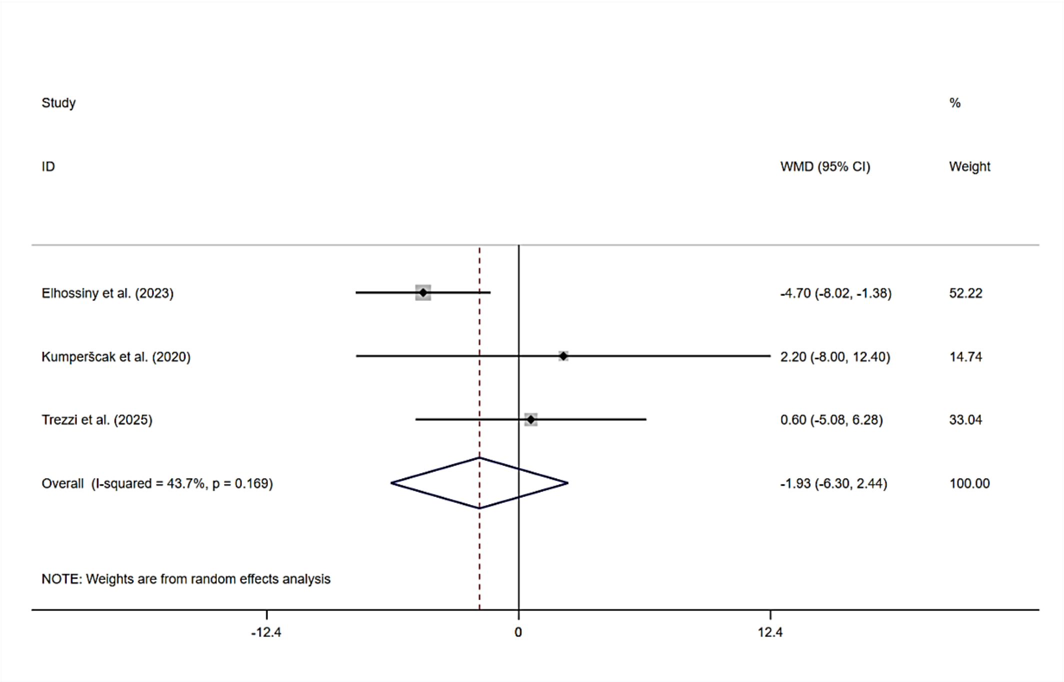

Figure S3. Forest plot of subgroup analysis for total scores on the CBCL.

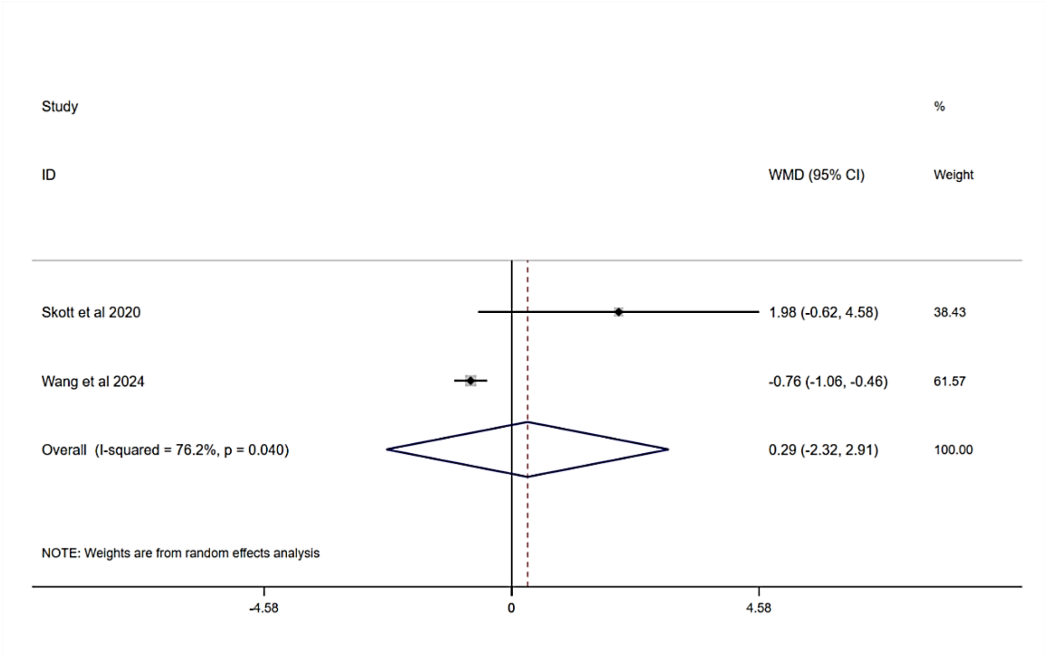

Figure S4. Forest plot of the SNAP-IV Inattention subgroup analysis.

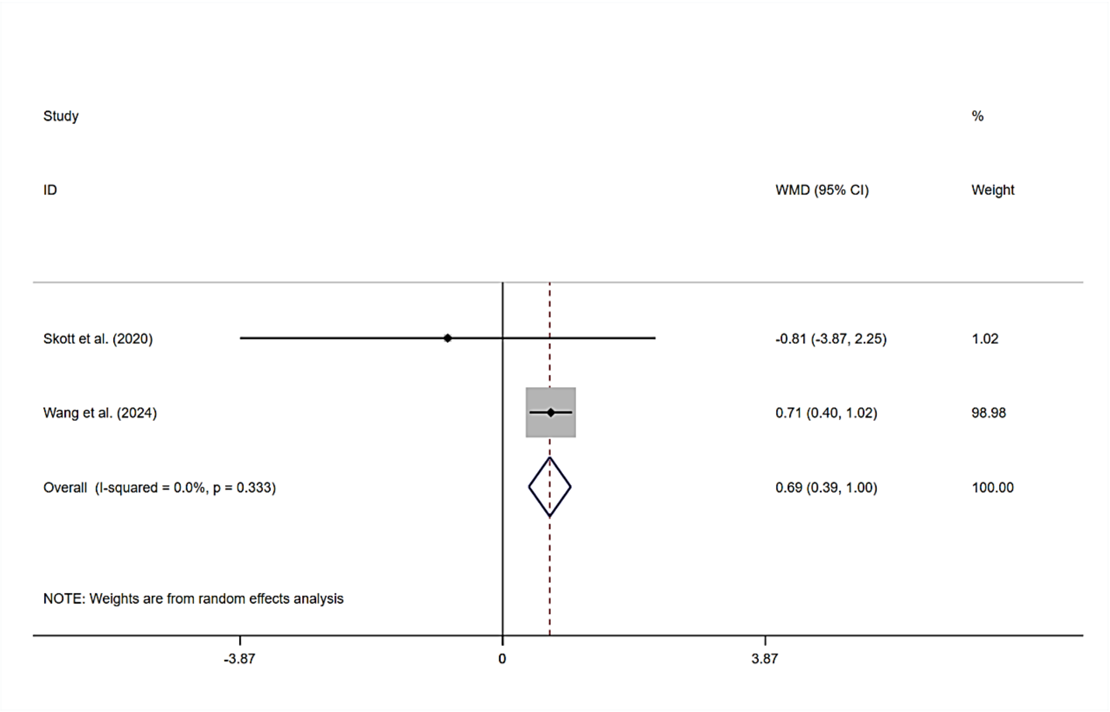

Figure S5. Forest plot of the subgroup analysis for SNAP-IV hyperactivity.

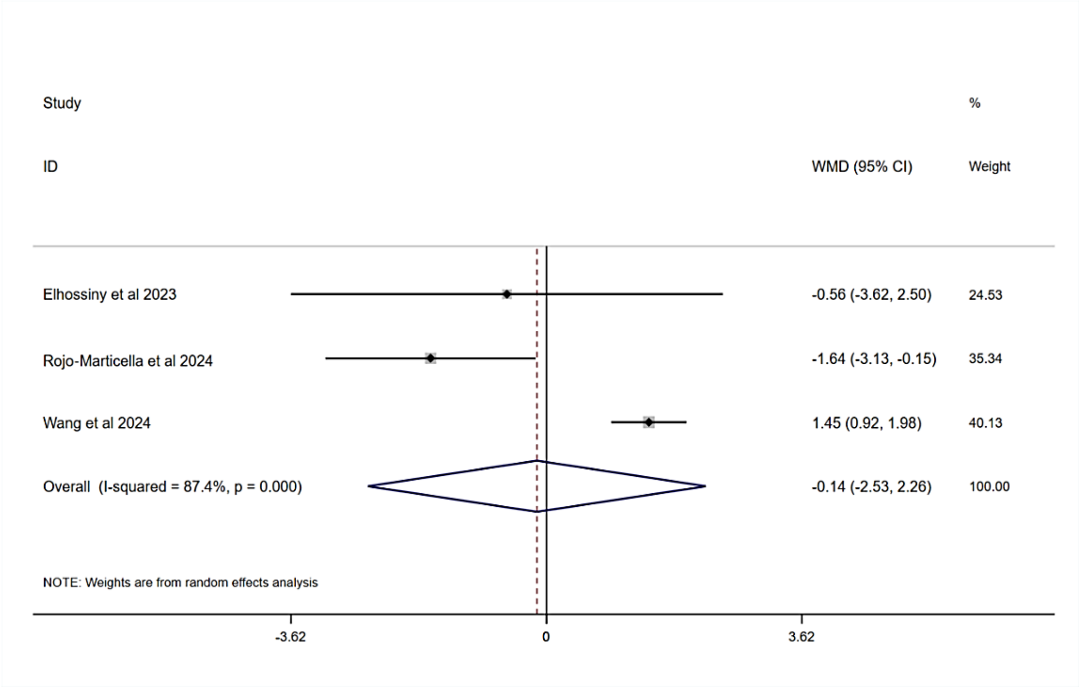

Figure S6. Forest plot for subgroup analysis of CPT commission errors.

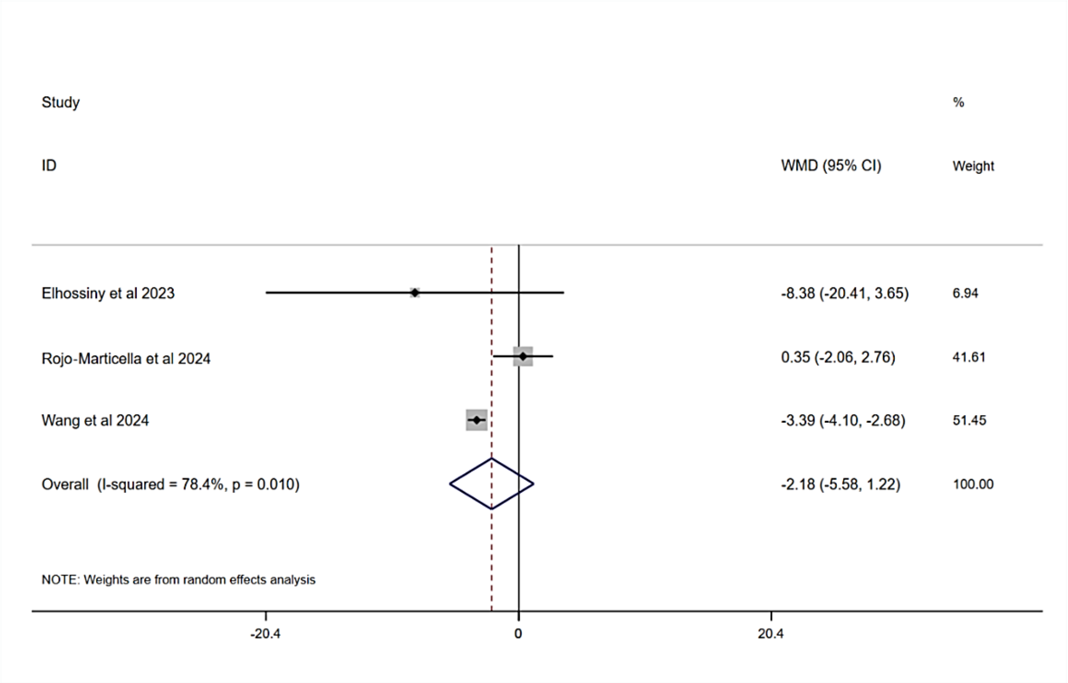

Figure S7. Forest plot for the subgroup analysis of CPT omission errors.

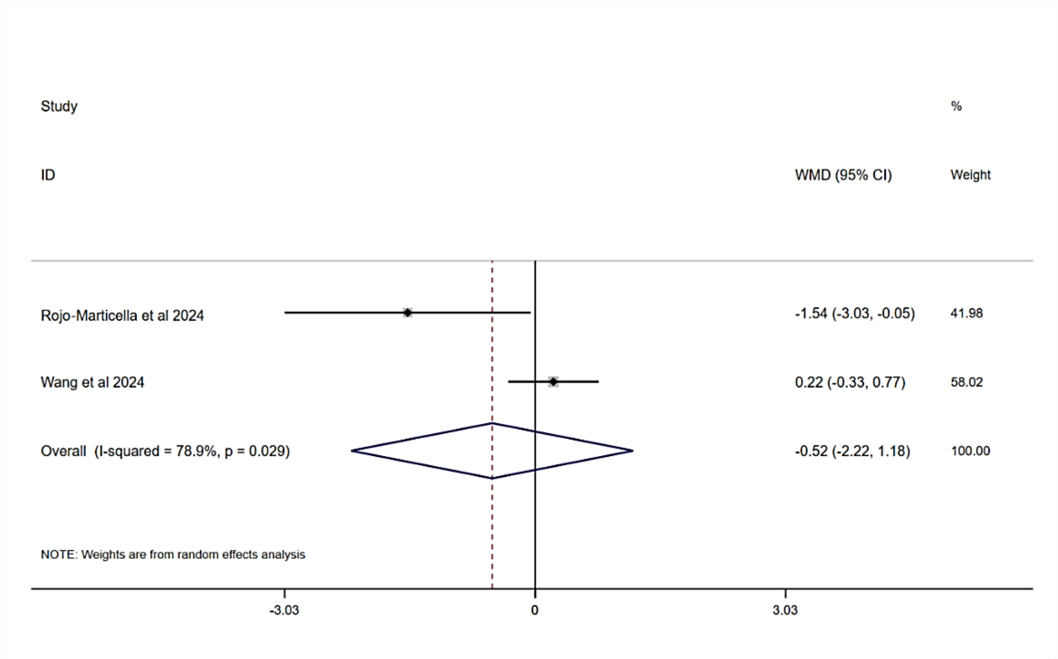

Figure S8. Forest plot for the subgroup analysis of CPT detectability.
